# Improving the Quantitative Basis of the Surgical Burden in Low-Income Countries

**DOI:** 10.1371/journal.pmed.1000149

**Published:** 2009-09-22

**Authors:** Theo Vos

**Affiliations:** University of Queensland, School of Population Health, Queensland, Australia

## Abstract

Theo Vos discusses how surgery is beginning to be considered an essential component of primary health care in low-income countries, and how we need to improve our understanding of the burden of surgical conditions in these settings.

For decades, the primary health care paradigm with a focus on maternal and child health programs has been driving funding for health in developing countries [1]. This has led to improvements, although there are doubts that many countries will meet the ambitious Millennium Development Goals 4 and 5 of reducing maternal and childhood mortality by three-quarters and two-thirds, respectively, between 1990 and 2015 [2],[3]. Surgery has not traditionally been considered an essential component of primary health care in low-income countries. This may be changing [4]. Emergency obstetric care, including surgical interventions, is now recognized as a key health service component for reducing maternal and neonatal mortality [5]. The broader contribution of surgical services to improvements in health outcomes in developing countries is also being discussed [6]–[9].

## Calculating the Burden of Surgical Conditions

A tentative estimate, described as the “best educated guess”, was that “surgical” disability-adjusted life years (DALYs) contribute 11% to the global burden of disease [7]. This figure is based on responses by 18 surgeons asked to estimate the proportion of each disease that would require surgery. After the two highest and two lowest estimates were discarded, the remaining 14 responses were averaged and then applied to Global Burden of Disease (GBD) 2002 disease estimates [10]. Injuries, cancers, congenital anomalies, obstetric complications, cataract, and perinatal conditions contributed 81% to the overall estimate of surgical DALYs globally. A limitation is that the question was asked only for conditions included in the GBD study. For example, most causes of intestinal obstruction, gall bladder disease, and inguinal hernia were omitted. Also, the role of circumcision in prevention of HIV infection was not considered at the time, as only recently has its efficacy become evident from trial data [11]. Inclusion of the latter, in particular, might have added considerably to the estimate.

There are multiple reasons to question the validity of the estimates for conditions that were included. One is an issue of definition. Debas et al. [7] defined a surgical condition as “any condition that requires suture, incision, excision, manipulation or other invasive procedure that usually, but not always, requires local, regional or general anesthesia.” The term surgical condition implies a dichotomy between surgical and nonsurgical conditions. While there are some conditions that could be classified as completely surgical (e.g., appendicitis or traumatic amputation of a leg) the reality is that many conditions may only partly be amenable to surgical intervention. Thus, it is better to define surgical services rather than surgical conditions. A similar pragmatic definition for surgical services would then be those services that “involve suture, incision, excision, manipulation or other invasive procedure that usually, but not always, require local, regional or general anesthesia.” It follows that the question to the experts should not have been “what, in your opinion, is the proportion of each condition that requires surgery?” but rather “what, in your opinion, is the proportion of mortality and disability from each condition that can be prevented or ameliorated by surgical services?”.

This is a complex question. The expert would need to make a judgment on the theoretical minimum burden for each condition if all in need had full access to the most efficacious surgical services. The approach is similar to that for calculating the proportion of disease burden that can be attributed to risk factors like tobacco, physical inactivity, or raised blood pressure. The difference between the current burden in DALYs for each condition and this theoretical minimum “counterfactual” [12] would constitute the “surgical burden.”

## Estimating Unmet Surgical Need

A variable proportion of surgical burden may already be met by current services and will not be included in burden of disease estimates. Burden of disease analysis is a cross-sectional snapshot of health loss in a population in a particular year, taking into account the “met need”—that is, health service action may already be preventing some deaths or cases of disease and disability. The burden of disease that is avertable by surgery would be the quantification of the “unmet need.” The potential health gain from surgical interventions is determined by trial data on efficacy. At the level of a population the total potential for health gain from surgery is the sum of the met and the unmet need. The unmet need is not only present in people who do not have access to surgical services but also includes the worse outcomes in people who receive less than optimal surgical care. The term “effective coverage” has been coined for the ratio of met need over the sum of met and unmet need [13]. Effective coverage of a health service or intervention is the proportion of potential health gain (i.e., that achieved if the service were delivered optimally to all those in need) achieved by current services as opposed to the surgical burden that remains in the population as a result of suboptimal use by those in need or suboptimal quality of delivery [14].

Let me illustrate this with a theoretical assumption of a condition X for which there is an efficacious surgical intervention that can reduce the disease burden by 80% (that is, the potential for health gain). However, due to limitations in the availability of trained staff, facilities, and resources, the effectiveness of the intervention reaches only 50% of its efficacy potential. Furthermore, only 50% of those in need have access to the services. If the current disease burden estimated for condition X is 100 DALYs, a theoretical back calculation of the disease burden in the absence of any treatment would be 100/(1−80%×50%×50%) = 125 DALYs. With full access and optimal surgical services, the theoretical minimum burden of condition X could be as low as 125×(1−80%) = 25 DALYs. Thus, the current surgical burden—the unmet need—is 100−25 = 75 DALYs. The met need is 125−100 = 25 DALYs.

For an accurate estimate of the overall surgical burden, this calculation needs to be replicated for each disease or consequence of disease that is amenable to surgery. What makes calculations even more difficult is that surgical interventions may have side effects and hence some of the health gain from its intended effect will be mitigated by the health loss from complications. For example, prostatectomy, even if carried out under optimal circumstances, carries a risk of impotence or incontinence.

The lack of existing evidence to support the required assumptions on efficacy, quality of intervention delivery, coverage, and potential harmful side effects means that we set experts an impossible task if we ask them to correctly integrate all this information into a single estimate of the surgical component of each disease and injury. The solution is to collect empirical evidence on each of these parameters and set a considerable research agenda to take the measurement of surgical burden forward.

Similar problems apply to the quantification of health outcomes in cost effectiveness studies of surgical interventions. Two case studies have indicated that the provision of surgical services in small hospitals in developing countries may rank among the most cost effective health service options [15],[16]. These studies estimated costs from empirical evidence, but health outcomes were based on expert opinion, similar to the studies quantifying the surgical burden. In fact, outcomes may be somewhat easier to estimate in this way, because in cost-effectiveness studies of a hospital's surgical services one is interested in the effectiveness (that is, efficacy modified by some factor to account for the quality of service delivery) and not in unmet need of cases not presenting. Even though surgical outcomes are more immediate and apparent than outcomes of medical or preventive interventions, two types of bias are likely to affect the opinions of surgeons. First, their experience may be dominated by short-term rather than long-term outcomes, and, second, their opinions may be biased toward the more severe cases they tend to see in specialist practice.

## More Data Needed to Estimate Surgical Outcomes

As with studies estimating surgical burden there is considerable scope for new data collection to improve the measurement of health outcomes in economic evaluations of surgical interventions. A logical expansion of the record review methods used in these two hospital case studies would be to collect evidence on the health outcomes of the surgical care at the time of discharge. Ideally, one would also like to evaluate longer-term outcomes to estimate the risks of permanent disability. In addition, the demographic surveillance systems such as those aligned under the INDEPTH Network [17] could be utilized to collect community-level information on the unmet need for surgical services.

The current interest in the role of surgery in international health will become more focused and its importance better recognised if efforts are made to improve the quantification of the health benefits brought about by surgical services. It is timely to start collecting this information, as greatly improved GBD estimates are currently being made with inputs from hundreds of experts worldwide [18]. As part of this update, the list of diseases for which estimates will be available has increased, allowing more accurate estimates of surgical burden of disease.

## Abbreviations

DALY: disability-adjusted life year
GBD: Global Burden of Disease
